# Enhancing LiDAR–IMU SLAM for Infrastructure Monitoring via Dynamic Coplanarity Constraints and Joint Observation

**DOI:** 10.3390/s25175330

**Published:** 2025-08-27

**Authors:** Zhaosheng Feng, Jun Chen, Yaofeng Liang, Wenli Liu, Yongfeng Peng

**Affiliations:** 1 China Harbour Engineering Company Limited, Beijing 100027, China; fengzs@chec.bj.cn; 2 China Communications Second Navigation Bureau First Engineering Co., Ltd., Wuhan 430416, China; chenj@chec.bj.cn; 3 School of Civil and Hydraulic Engineering, Huazhong University of Science and Technology, Wuhan 430074, China; m202271514@hust.edu.cn (Y.L.); liu_wenli@hust.edu.cn (W.L.)

**Keywords:** SLAM, error-state Kalman filter, LiDAR, LiDAR–IMU fusion, pose estimation

## Abstract

Real-time acquisition of high-precision 3D spatial information is critical for intelligent maintenance of urban infrastructure. While SLAM technology based on LiDAR–IMU sensor fusion has become a core approach for infrastructure monitoring, its accuracy remains limited by vertical pose estimation drift. To address this challenge, this paper proposes a LiDAR–IMU fusion SLAM algorithm incorporating a dynamic coplanarity constraint and a joint observation model within an improved error-state Kalman filter framework. A threshold-driven ground segmentation method is developed to robustly extract planar features in structured environments, enabling dynamic activation of ground constraints to suppress vertical drift. Extensive experiments on a self-collected long-corridor dataset and the public M2DGR dataset demonstrate that the proposed method significantly improves pose estimation accuracy. In structured environments, the method reduces z-axis endpoint errors by 85.8% compared with Fast-LIO2, achieving an average z-axis RMSE of 0.0104 m. On the M2DGR Hall04 sequence, the algorithm attains a z-axis RMSE of 0.007 m, outperforming four mainstream LiDAR-based SLAM methods. These results validate the proposed approach as an effective solution for high-precision 3D mapping in infrastructure monitoring applications.

## 1. Introduction

With the ongoing expansion of urban infrastructure, the efficient and accurate acquisition of its three-dimensional spatial information has become a critical factor in promoting intelligent upgrades in urban infrastructure maintenance [1]. Traditional manual inspection-based maintenance models are facing efficiency bottlenecks, as intelligent mobile robot systems, leveraging their autonomy and all-weather operational capabilities, are gradually becoming the core tools for monitoring the full lifecycle of infrastructure. In this context, simultaneous localization and mapping (SLAM) technology, based on the fusion of heterogeneous sensors such as LiDAR and inertial measurement units (IMU), can construct high-precision three-dimensional spatial representations of dynamic environments. The localization accuracy and map quality of SLAM directly impact the reliability of geometric modeling, thereby offering a theoretical and technical framework for the digital transformation of urban infrastructure maintenance [2].

However, mainstream SLAM algorithms inevitably suffer from pose estimation drift, whether they are based on nonlinear optimization [3] or Kalman filtering [4]. In six-degree-of-freedom (6-DoF) pose estimation tasks dominated by LiDAR sensors, the drift issues along the roll, pitch, and z-axes are particularly pronounced. This phenomenon stems from two critical factors. First, data-acquisition robots typically mount mechanical LiDARs (e.g., Velodyne VLP-16) in horizontal configurations, resulting in inherently sparse vertical measurements. For instance, the VLP-16 sensor has a vertical resolution of only 2° and a limited vertical field of view (FoV) of 30°, leading to insufficient geometric constraints for vertical-axis pose estimation in SLAM systems. Second, according to J. Laconte et al. [5], LiDAR measurement biases caused by large incident angles at ground regions further degrade ranging accuracy. As incident angles increase, the measurement precision deteriorates systematically, causing cumulative errors during point cloud registration. This error propagation ultimately manifests as significant pose estimation drift.

In urban infrastructure scenarios dominated by rigid structures, ground regions typically exhibit strong coplanarity within local areas. Capitalizing on this attribute to address the aforementioned challenges, this paper presents a multi-sensor fusion localization and mapping algorithm based on an improved error-state Kalman filter, designed for application on data acquisition robots in infrastructure maintenance scenarios. Specifically, our contributions include the following:

- Design of a ground point cloud extraction algorithm based on angular thresholding, which effectively distinguishes ground from non-ground points through vertical angular analysis of LiDAR point clouds frame-by-frame. This significantly enhances the accuracy and robustness of ground point cloud extraction, thereby improving practical applicability in engineering applications.
- Development of a ground constraint module that exploits the local planar consistency prior inherent in urban infrastructure environments, incorporating coplanarity assessment functionality. By conditionally activating ground constraints, this approach effectively filters outliers, mitigates pose estimation drift, and enhances system robustness and reliability.
- Integration of ground constraints with traditional LiDAR point cloud registration constraints through joint optimization to obtain optimal pose estimates, enabling construction of high-precision point cloud maps.

The remainder of this article is organized as follows. Section 2 reviews relevant academic research. Section 3 presents the proposed system. Section 4 analyzes and discusses the experimental results. Finally, Section 5 summarizes the entire text and outlines future research directions.

## 2. Related Work

Since its conceptual introduction at the 1986 IEEE International Conference on Robotics and Automation (ICRA) [6], SLAM algorithms have evolved into two major technical paradigms: filter-based and optimization-based approaches. In this section, we provide a brief review of the academic research related to these two paradigms.

### 2.1. Filter-Based SLAM Approaches

Filtering-based SLAM algorithms emerged during the formative years of SLAM technology. In the era of limited arithmetic power, this type of method became mainstream due to its high computational efficiency, and its core idea is to estimate the amount of state by recursion, for example, using the state of the previous moment to deduce the state of the next moment. Early SLAM research primarily focused on filter frameworks, commonly employing the extended Kalman filter (EKF) to solve state estimation problems in SLAM [7]. In 2001, J. Neira et al. demonstrated the sensitivity of the EKF algorithm to prediction association errors, highlighting the limitations of linearization assumptions in motion and observation models [8]. To overcome this bottleneck, Montemerlo et al. proposed FastSLAM [9], which combined particle filtering with Bayesian estimation for the first time, enhancing robustness in nonlinear scenarios by decoupling state estimation from map updates. Its improved version, FastSLAM 2.0 [10], further incorporated EKF-based pose updates, effectively mitigating drift caused solely by kinematic recursion. Recently, Qin et al. introduced LINS [4], a lightweight laser-IMU fusion framework utilizing the error-state Kalman filter (ESKF) to significantly reduce computational costs, marking a breakthrough in lightweight SLAM system development.

Filter-based SLAM approaches update system states recursively, offering low-latency performance but remaining susceptible to accumulated errors. This limitation has driven researchers toward optimization-based methods with global perspective advantages.

### 2.2. Optimization-Based SLAM Approaches

In the modern SLAM period, optimization methods have become mainstream, especially for applications in vision SLAM. Optimization methods bundle the globally accumulated information into an offline estimation of the robot’s entire trajectory and waypoints. Thanks to the increasing maturity of computer vision research and the significant improvement of computer performance, optimization methods based on visual sensors have become the mainstream direction of modern SLAM research. In 1997, Lu et al. formulated the SLAM problem as a maximum a posteriori probability estimation through graph optimization theory [11], marking the emergence of optimization methods by reducing trajectory drift through historical sensor data fusion. Subsequently, Gutmann et al. proposed an efficient loop closure detection method, establishing an incremental graph optimization-based SLAM framework [12]. Kschischang et al. introduced factor graph models to infer intrinsic variable dependencies, where each factor serves as a constraint relative to system poses, advancing optimization methods through constraint relationships among variables [13]. Thereafter, optimization-based approaches experienced sustained development, gradually forming modern optimization-centric SLAM architectures [14,15,16].

In this context, significant progress has also been made in a series of optimization methods using LiDAR as the main sensor. LOAM [17] is a classical real-time LiDAR odometry and map-building method, which decomposes the complex SLAM problem into two algorithms running in parallel: a high-frequency, low-accuracy odometry estimation algorithm and a low-frequency, high-accuracy map-building algorithm. This divide-and-conquer strategy effectively balances computational efficiency and accuracy. To cope with the localization challenges in large-scale environments, SegMap [18] proposes a map representation method based on 3D point cloud segmentation. The method achieves robust relocation and closed-loop detection by extracting structured segments in the scene and matching them using data-driven descriptors.

To enhance map density while achieving real-time mapping operations, SuMa [19] adopts a surfel-based map representation and realizes fast alignment of the current frame to the map model by correlating the projected data. In addition, SuMa utilizes the rendered virtual map view for closed-loop inspection and validation to build globally consistent dense maps. For dynamic urban scenes, SuMa++ [20] introduces semantic information on top of SuMa. By extracting the semantic labels of point clouds through a fully convolutional neural network, SuMa++ can effectively filter out dynamic objects and utilize semantic constraints to improve the robustness of position estimation, and ultimately generate dense 3D maps with rich semantic information.

Introduced in 2016, Google’s Cartographer algorithm [21] integrated laser scan matching within submaps, loop closure detection, and graph optimization, emerging as a cornerstone 2D SLAM solution widely deployed in engineering practice. LeGO-LOAM [22], proposed by Tixiao Shan et al., achieved efficiency–accuracy balance in complex terrains through a two-step optimization strategy leveraging ground and corner constraints. With growing multi-sensor fusion demands, the authors of LeGO-LOAM subsequently developed LIO-SAM [23] and LVI-SAM [24], both of which construct pose constraints using factor graphs. The latter augmented LIO-SAM with visual data via a tightly coupled architecture that merged LiDAR-inertial and visual-inertial subsystems. Koide et al. proposed hdl_graph_slam [3], innovatively fusing multi-source constraints including GPS, ground planes, LiDAR odometry, and loop closure constraints to suppress long-term drift errors. Recently, Lin et al. introduced BALM [25], a SLAM framework that incorporated bundle adjustment (BA), a technique from visual SLAM, into LiDAR-based SLAM, effectively mitigating localization drift. Xu’s team developed Fast-LIO [26] and Fast-LIO2 [27], where Fast-LIO utilized ESKF for tight integration of LiDAR and IMU data to reduce mapping errors, while Fast-LIO2 introduced an incremental k-d tree for global map maintenance alongside direct point cloud-map registration. Lin et al. further proposed R3LIVE [28], a multi-sensor fusion system combining LiDAR, inertial measurements, and visual camera data to achieve real-time localization, mapping, and colorization. Zheng’s team advanced Fast-LIVO2 [29] through ESKF-based efficient fusion of IMU, LiDAR, and image measurements, employing a sequential update strategy to address synchronization challenges in heterogeneous sensor networks. In recent years, researchers have proposed several innovations to address the challenges of LiDAR–IMU fusion algorithms. To tackle the vertical, pose drift problem, SDV-LOAM [30] introduced an adaptive optimization strategy that dynamically adjusts the degrees of freedom based on geometric constraints, effectively suppressing the drift. Performance in challenging environments has also been a key focus. To improve performance in feature-sparse environments, D-LIOM [31] adopted a tightly-coupled direct method, enhancing efficiency and robustness by directly registering raw point clouds. For scenarios with aggressive and unsmooth motion, such as those encountered by quadruped robots, Zhou et al. [32] proposed a tightly coupled SLAM algorithm using a Normal Distribution Transform (NDT)-based registration method to improve robustness. Furthermore, fusing different sensor modalities has become an effective approach to boost system performance. One strategy involves integrating heterogeneous LiDARs; for instance, Li et al. [33] developed a system that fuses the wide field of view (FoV) of a spinning LiDAR with the dense, high-resolution measurements of a solid-state LiDAR to achieve both robust ego-estimation and detailed maps. A more common approach is the fusion of visual information with LiDAR-inertial data. LVIO-fusion [34], for example, achieves high-precision state estimation in degenerate environments, while R3LIVE++ [35] combines state estimation with radiance map reconstruction, demonstrating the latest advancements. To handle the complexities of such fusion, recent works have targeted specific challenges. In dynamic environments, frameworks like LVI-fusion [36] and another by Cai et al. [37] incorporate object-detection networks to identify and remove transient objects such as pedestrians and vehicles, which significantly improves mapping quality. Additionally, to achieve a more robust fusion, LVI-fusion also proposes a method to assign reliable depth to visual features using a local LiDAR point cloud map. Despite these advancements, existing methods still face challenges, including insufficient utilization of geometric priors, misalignment of dynamic scene data, and computational efficiency bottlenecks.

Although filter-based SLAM approaches and optimization-based SLAM approaches have achieved significant progress, current localization and mapping methodologies share common limitations. Most studies underutilize structural geometric information in scenes, with existing algorithms lacking sufficient positioning accuracy for urban infrastructure scenarios. Future research demands scene geometry-aware multi-sensor fusion strategies for enhanced localization and mapping performance.

## 3. Proposed Method

**Figure 1:**
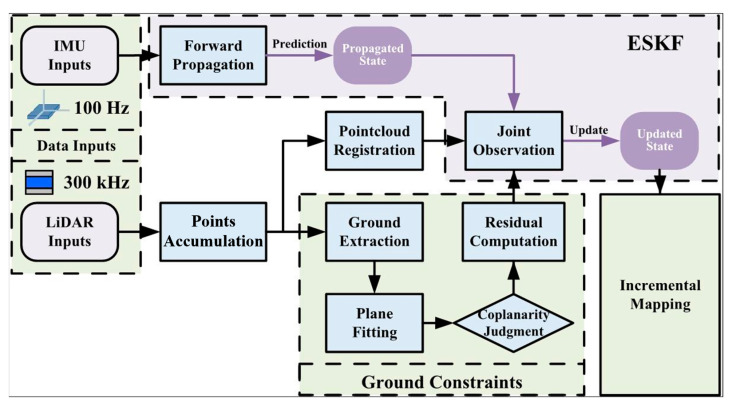
Framework diagram of a radar-inertial odometry algorithm based on improved FAST-LIO2.

This section details the LiDAR-inertial odometry framework developed in this study. The framework is built upon and deeply improves the advanced FAST-LIO2 algorithm. First, in Section 3.1, we provide an overview of the core architecture of FAST-LIO2, which serves as the theoretical foundation for the subsequent improvements. Subsequently, in Section 3.2, inspired by and optimized from the ground point cloud extraction methodology in LeGO-LOAM, we design a more robust ground segmentation algorithm. Next, to mitigate the drift in pose estimation, under the assumption of local ground plane consistency, we develop a ground constraint module in Section 3.3. Finally, in Section 3.4, we integrate this ground constraint with the inherent LiDAR point cloud registration constraints of FAST-LIO2 into a unified optimization framework, forming a novel joint observation model. This results in the final improved SLAM algorithm framework (improved FAST-LIO2 LiDAR SLAM). The overall system architecture is illustrated in Figure 1.

### 3.1. Overview of FAST-LIO2

FAST-LIO2, as an advanced LiDAR-inertial odometry system, is fundamentally built upon an error-state Kalman filter framework, enabling tightly coupled multi-sensor data integration. Compared to conventional solutions, the algorithm achieves significant performance improvements through two breakthrough design innovations: first, it introduces an incremental dynamic indexing structure (ikd-Tree) for map management, enabling millisecond-level node insertion, deletion, and topological restructuring, effectively addressing the challenge of real-time map updating in large-scale environments; second, by abandoning traditional preprocessing pipelines reliant on handcrafted feature extraction, it employs a raw point cloud matching strategy that preserves high-dimensional geometric information within the environment, providing richer constraints for pose estimation.

From an engineering perspective, this framework demonstrates three notable advantages: (1) computational efficiency—within outdoor environments spanning hundreds of meters, the system achieves real-time pose tracking and 3D mapping at up to 100 Hz; (2) environmental adaptability—the framework has been validated under extreme motion scenarios with angular velocities exceeding 1000 degrees per second, confirming its strong robustness in complex dynamic environments; (3) measurement accuracy—in evaluations across multiple public datasets, its localization and mapping precision consistently outperforms various current state-of-the-art approaches. Importantly, these performance gains are achieved without reliance on hardware acceleration or distributed computing architectures, underscoring the superiority of its algorithmic design.

Given the outstanding performance and robust architecture of FAST-LIO2, this study adopts it as the foundational framework. Targeting typical urban infrastructure environments characterized by rich planar features, we conduct in-depth algorithmic optimization and functional enhancement.

### 3.2. Ground Extraction

To effectively establish ground constraints within a LiDAR-inertial odometry (LIO) framework, it is essential to accurately and robustly segment ground points from each LiDAR scan. This study proposes an improved ground extraction algorithm inspired by the ground segmentation principle of the LeGO-LOAM framework [16], enhanced through the introduction of a multi-stage outlier removal strategy. This addresses the issue of false positives and missed detections commonly encountered by conventional methods in complex environments.

For mechanical spinning LiDAR sensors mounted in a horizontal orientation—such as the Velodyne VLP-16—the ground point cloud exhibits the following characteristics in the LiDAR coordinate system: (1) z-coordinates are predominantly low; (2) the points form a concentric circular pattern; and (3) locally, they can be approximated as planar surfaces. Based on these observations, the algorithm identifies ground points by examining vertical angle differences between point pairs from adjacent scan lines within the same horizontal azimuth.

Specifically, for each pair of points from adjacent scan lines sharing the same horizontal direction, the vertical angle between them is computed as:

(1) $$\theta =\arctan\left(\frac{∆z}{\sqrt{∆x^{2}+∆y^{2}}}\right)\leq \varepsilon_{\theta }$$

where ∆x, ∆y, ∆z represent the coordinate differences between qualifying point pairs, and $\varepsilon_{\theta }$ denotes a predefined angular threshold. Point pairs satisfying the above inequality are classified as belonging to the ground region. By iterating through all points and evaluating this condition, continuous clusters of points that meet the angular constraint are extracted as candidate ground point sets.

In practical scenarios, LiDAR point clouds are susceptible to interference from dynamic obstacles, sensor noise, and self-occlusion. To enhance the robustness of the algorithm, a three-stage outlier suppression strategy is designed:

- Dynamic search truncation: Points that are too close to or too far from the LiDAR center (<0.3 m or >50 m) are skipped to avoid ego-body interference and long-range measurement noise.
- Cross-obstacle detection: A radial distance ratio threshold $\varepsilon_{\sigma }$ is introduced. When the ratio of radial distances between adjacent points exceeds this threshold, the pair is considered to span an obstacle, and the current column search is terminated, as formulated below:
  (2) $$\sigma =\frac{\max\left(d_{1},d_{2}\right)}{\mathrm{mix}\left(d_{1},d_{2}\right)}\leq \varepsilon_{\sigma }$$

- Invalid point tolerance: Points with NaN (Not a Number) values are automatically skipped to prevent computational failures during processing.

### 3.3. Ground Constraint

In urban infrastructure environments dominated by rigid structures, the ground typically exhibits a high degree of local planarity. To exploit this characteristic, this study introduces the local ground plane consistency assumption, which models the ground as an infinitely extended plane with fixed slope and height within local regions during the SLAM process. By integrating real-time ground observations with historical plane parameters, this constraint effectively suppresses pose estimation drift. The key mathematical formulation is as follows.

#### 3.3.1. Plane Parameterization and Residual Definition

Let the plane parameters fitted from the ground point cloud in a given LiDAR scan be denoted as $\pi ={\left(n^{T},d\right)}^{T}\in R^{4}$, where n represents the normalized normal vector of the plane, and d is the intercept, indicating the distance from the origin to the plane along the normal direction. The local dominant plane parameters $\pi_{m}$ are calibrated at the initial stage within the local region. The parameters of the initial plane are obtained by fitting the ground point cloud from the first radar frame at the initial time to a plane. The process of plane fitting is as follows:

Given a set of radar point clouds consisting of *N* three-dimensional spatial points, their three-dimensional Euclidean coordinates are represented as $P_{k}=\left(x_{k},y_{k},z_{k}\right),k=1,2,\ldots ,N$. Find a suitable set of plane parameters (n,d) to construct the following least squares problem:

(3) $$\underset{\;n,d}{min}\sum_{k=1}^{N}{\left\|n^{T}p_{k}+d\right\|}_{2}^{2}$$

where $n\in R^{3}$ is the normalized normal vector, and d∈R is the intercept.

The ground constraint residual is then defined as:

(4) $$r_{g}=\pi_{c}-\pi_{m}^{L_{t}}$$

where $\pi_{c}$ denotes the ground plane parameters at the current time step, and $\pi_{m}^{L_{t}}$ represents the result of transforming the initial plane parameter $\pi_{m}$ into the current LiDAR coordinate frame $L_{t}$.

#### 3.3.2. Coordinate Transformation and Observation Model

**Figure 2:**
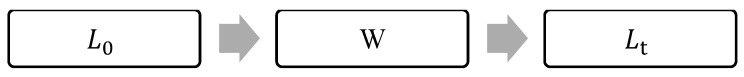
Three-stage coordinate transformation chain for ground constraint enforcement in GC-LIO.

To realize the real-time computation of $\pi_{m}^{L_{t}}$, a three-stage coordinate transformation chain is constructed for $\pi_{m}$ (as illustrated in Figure 2):

1. Initial calibration:

In the initial LiDAR coordinate frame $L_{0}$, the dominant plane $\pi_{m}^{L_{0}}$ is fitted;

2. Transformation to world coordinate system:

(5) $$\pi_{m}^{W}=\left[\begin{array}{c} n_{m}^{W}\\ d_{m}^{W} \end{array}\right]=\left[\begin{array}{c} R_{I_{0}L_{0}}\cdot n_{m}^{L_{0}}\\ d_{m}^{L_{0}}-{\left(R_{I_{0}L_{0}}{\;n}_{m}^{L_{0}}\right)}^{T}\cdot t_{I_{0}L_{0}} \end{array}\right]$$

where $R_{I_{0}L_{0}}$ and $t_{I_{0}L_{0}}$ denote the extrinsic rotation and translation from the LiDAR to the IMU at the initial time;

3. Transformation to current frame LiDAR coordinate system:

(6) $$\pi_{m}^{L_{t}}=\left[\begin{array}{c} R_{I_{t}L_{t}}^{T}R^{T}\cdot n_{m}^{W}\\ d_{m}^{W}+{\left(n_{m}^{W}\right)}^{T}t+{\left(n_{m}^{W}\right)}^{T}Rt_{I_{t}L_{t}} \end{array}\right]$$

where $R_{I_{t}L_{t}}$ and $t_{I_{t}L_{t}}$ represent the extrinsic parameters between the LiDAR and IMU at the current time; and R and t denote the current system pose.

Thus, the nonlinear observation equation is established:

(7) $$\left\{\begin{array}{c} z_{g}=h\left(x\right)+v_{g}\\ h\left(x\right)=\pi_{m}^{L_{t}} \end{array}\right.$$

where x is the system state to be estimated; h(x) is the observation function; and $v_{g}\sim N(0,V)$ is the observation noise.

#### 3.3.3. Transformation to Current Frame LiDAR Coordinate System

Given the geometric characteristics of the planar constraint, the ground constraint can only restrict the pitch angle, roll angle, and normal translation (z), but it cannot observe the yaw angle or horizontal translations (x, y). Therefore, the corresponding dimensions in the Jacobian matrix $H=\frac{\partial h}{\partial \delta_{x}}$ need to be explicitly set to zero to ensure the physical validity of the optimization problem.

Additionally, the ground constraint relies on the assumption of local ground planar consistency, which assumes that the current ground plane is the same as the main plane at the current time step. To avoid incorrect constraints, a dual coplanarity check condition is introduced:

(8) $$\left\{\begin{array}{c} \theta =\arccos\left(\frac{n_{c}^{T}n_{m}^{L_{t}}}{\left|n_{c}\right|\left|n_{m}^{L_{t}}\right|}\right)\leq \varepsilon_{\theta }\\ ∆d=\left|d_{c}-d_{m}^{L_{t}}\right|\leq \varepsilon_{d} \end{array}\right.$$

The ground constraint is only activated when both of the above conditions are satisfied simultaneously.

### 3.4. Joint Observation

To address the weak horizontal pose constraints provided by ground point clouds, this paper proposes a differentiated processing strategy: ground point clouds are specifically used for planar constraint observations (Section 3.3), while non-ground point clouds participate in point cloud registration. The two types of constraints are fused through a joint observation model as follows:

(9) $$h=\left(\frac{h_{icp}}{h_{g}}\right)$$

Specifically, $h_{icp}$ denotes the observation model corresponding to point cloud registration, while $h_{g}$ denotes the observation model associated with the ground constraint.

Due to the difference in noise characteristics between the ground constraints and point cloud registration, the Kalman gain computation method used in FAST-LIO2 cannot be directly applied. To address this, an improved gain is derived based on the principle of block matrices:

(10) $$K={\left(\frac{H_{icp}^{T}H_{icp}}{n_{icp}}+\frac{H_{g}^{T}H_{g}}{n_{g}}+{\overset{ˇ}{P}}^{-1}\right)}^{-1}\cdot \left[\frac{H_{icp}^{T}}{n_{icp}}\vdots \frac{H_{g}^{T}}{n_{g}}\right]$$

Among them, $H_{icp}$ and $n_{icp}$ denote the H matrix and noise coefficient for the point-to-plane ICP component of point cloud registration, while $H_{g}$ and $n_{g}$ represent the H matrix and noise coefficient for the ground constraint component, respectively. This formulation avoids inverting high-dimensional matrices, thereby preserving the computational efficiency advantages of Fast-LIO2.

So far, the system’s posterior error state has been optimally updated, completing one iteration. Finally, the distortion-corrected feature points are projected into the world coordinate system based on the optimized pose, enabling incremental updates to the global map.

In summary, building upon FAST-LIO2 and incorporating improvements through three key modules—ground segmentation, ground constraint, and joint observation—we propose a SLAM system that achieves significantly enhanced localization accuracy and mapping quality in typical planar scenarios such as urban infrastructure environments.

## 4. Experiment and Results

This section conducts a comprehensive experimental evaluation of the proposed GC-LIO algorithm. To thoroughly validate its performance and robustness, we designed two distinct sets of experiments. First, to address the need for repeatable, small-scale validation in structured environments, we conducted tests using a self-built mobile robot platform on a self-collected dataset from a long corridor scene. Second, to demonstrate the algorithm’s accuracy and mapping capabilities in large-scale, complex scenarios, we utilized the public M2DGR dataset [38]. In Section 4.1, we will introduce the hardware platforms and sensor configurations for both experimental setups. Then, Section 4.2 defines the error metrics used for performance evaluation. Finally, in Section 4.3, we will show and discuss the experimental results in detail, including accuracy analysis and ablation studies.

### 4.1. Experimental Setups

This section will provide a detailed overview of the hardware platform and sensor configuration used in the experiment. Additionally, prior to the experiment, we determined the six-degree-of-freedom external transformation between the LiDAR and IMU for both the self-built platform and the M2DGR dataset platform. We adopted the open-source LI-Calib toolbox [39], which is based on a continuous-time batch estimation method for target-free calibration. This method achieves joint optimization by simultaneously minimizing the residuals between the raw IMU measurements and the laser radar point-to-surface distance in a B-spline parameterized trajectory. This target-free method is highly suitable for practical deployment and can accurately handle high-rate asynchronous data. The calibration sequence was executed under sufficient rotational and translational excitation to ensure observability, and the obtained external parameters were kept fixed in all subsequent experiments.

#### 4.1.1. Self-Built Platform and Self-Collected Dataset

**Figure 3:**
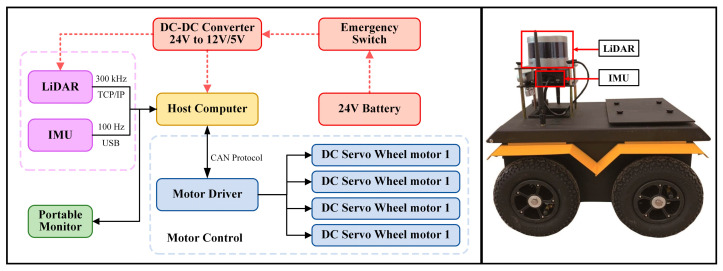
Robotic system architecture overview, including data flow (**left**) and robot schematic (**right**).

The mobile robot system platform used in this study is shown in Figure 3. This platform integrates four core modules: mechanical structure, actuation, control, and sensing. At the perception level, the robot is equipped with a LiDAR (Light Detection and Ranging) sensor and an Inertial Measurement Unit (IMU), which serve as the primary sensors for localization and mapping. The LiDAR has a data output rate of approximately 300 kHz and communicates with the main computer via the TCP/IP protocol. The IMU operates at a frequency of 100 Hz and interfaces with the host through USB. Detailed specification parameters of the LiDAR and IMU are listed in Table 1 and Table 2, respectively.

Table 1 lists the key specification parameters of the LiDAR used in this study. The LiDAR model features a 360° horizontal field of view (FoV), enabling comprehensive, blind-spot-free perception of the surrounding environment. It has a ranging accuracy of ±3 cm and a maximum detection range of up to 100 m, which is sufficient for mapping large-scale environments. Notably, its vertical field of view is 30° with a vertical angular resolution of 2°. This relatively sparse scanning pattern in the vertical direction is one of the key factors contributing to vertical drift in traditional SLAM algorithms, highlighting the necessity of the ground constraint method proposed in this paper.

Table 2 presents the specification parameters of the IMU. The IMU supports a maximum sampling rate of 1 kHz, allowing it to provide high-frequency measurements of angular velocity and acceleration. Its accelerometer and gyroscope offer resolutions of 0.001 g and 0.001°/s, respectively. The high-precision IMU data serves as the foundation for high-frequency motion prediction within the ESKF framework, offering reliable support for accurate pose estimation during the low-frequency scanning intervals of the LiDAR.

#### 4.1.2. Public M2DGR Dataset

**Figure 4:**
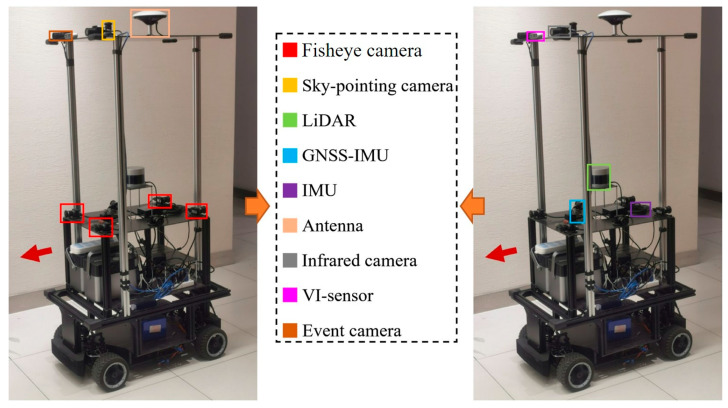
M2DGR multi-sensor fusion mobile robot platform.

To evaluate the localization accuracy advantage of the proposed method, this paper uses the Hall04 and Street04 sequences from the publicly available dataset M2DGR for testing. The M2DGR dataset is captured by a set of multi-sensor fusion mobile robot platform that integrates a variety of sensing sensors. The core sensing devices of the platform and their mounting positions on the robot are shown in Figure 4, including LiDAR, IMU, GNSS-IMU, fisheye camera, infrared camera, event camera, VI sensor, etc. The detailed parameters of various sensors are shown in Table 3. Among them, LiDAR and IMU are used as the main sensors of the SLAM algorithm, and their high-precision data provide the basis for this study.

The sensors of the platform are distributed in two layers, where the LiDAR is located in the upper part of the lower layer and is capable of providing 360° panoramic scanning for generating highly accurate 3D point cloud maps. Inertial sensors such as IMU and GNSS-IMU, on the other hand, provide high-frequency motion information to support position estimation. In addition, a variety of cameras (e.g., fisheye, infrared, and event cameras) integrated on the platform provide visual information for future studies, but in this paper, we mainly utilize LiDAR and IMU data for experiments.

Among them, the 360° horizontal FoV of the Velodyne VLP-32C LiDAR is able to realize a comprehensive perception of the surrounding environment, and its maximum detection distance is up to 200 m, which is sufficient to meet the needs of large-scale environment mapping. It is worth noting that its vertical FoV ranges from −30° to +10°, and the vertical angular resolution is relatively sparse, which is one of the key factors of the vertical drift problem in traditional SLAM algorithms, thus highlighting the necessity of the ground constraint method proposed in this paper. The high-frequency motion measurements provided by sensors such as GNSS-IMU, IMU, etc., provide reliable support for high-frequency motion prediction in the ESKF framework, which ensures accurate position estimation during the LiDAR low-frequency scanning interval.

### 4.2. Evaluation Metrics and Baseline Algorithms

This study used absolute position error (APE) as the primary evaluation metric in both datasets. APE serves as the quantitative performance metric for evaluating SLAM algorithms in this study, it is a widely used metric. APE measures the global trajectory accuracy by aligning the algorithm-estimated trajectories with the true-value trajectories provided by the dataset, and then calculating the difference between the corresponding bit position points. APE is precisely defined as the root mean square error (RMSE) across all trajectory points. The calculation formula is as follows:

(11) $$APE=\sqrt{\frac{1}{m}\sum_{j=1}^{m}{\left({\hat{T}}_{j}-T_{j}\right)}^{2}}$$

**Figure 5:**
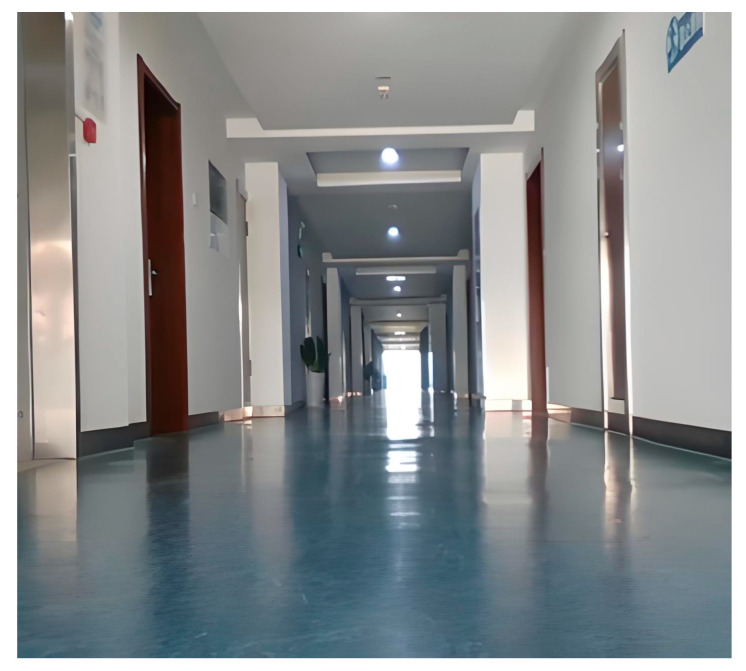
Scene diagram of a long corridor.

For the corridor dataset collected independently, in order to verify its effectiveness in improving positioning accuracy, this section uses its baseline algorithm, Fast-LIO2, for direct comparison. The dataset is based on a long corridor in an office. During the data collection process, the robot made two turns and eventually returned to the starting point via the same route. The experimental scenes are shown in Figure 5.

**Figure 6:**
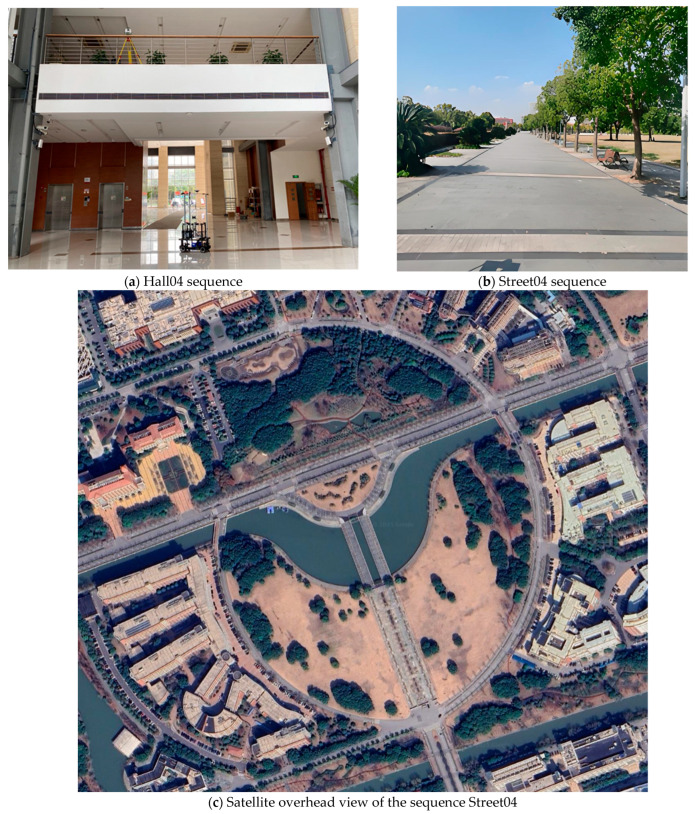
Algorithm test scenarios for the M2DGR dataset.

For the M2DGR dataset, the Hall04 and Street04 sequences are adopted to evaluate the localization accuracy and map-building performance of GC-LIO against multiple mainstream SLAM algorithms. The Hall04 sequence is captured in an indoor hall with a large flat floor, which is an ideal scenario to examine the validity of the ground constraint. The Street04 sequence is collected in a campus road environment containing slopes and more complex structures, and is used to test the robustness and mapping performance in a real urban scenario. The experimental scenes are shown in Figure 6.

In the M2DGR dataset, we compared the proposed algorithm with four representative open-source SLAM algorithms based on lidar, which are the following:

- A-LOAM [17]: A-LOAM is a code implementation and optimized version of the original LOAM algorithm, which mainly improves the readability and implementation efficiency of the code;
- LeGO-LOAM [22]: A lightweight LiDAR odometry and mapping algorithm optimized for ground-based applications;
- LIO-SAM [23]: A tightly coupled LiDAR-inertial odometry approach based on smoothing and mapping;
- Fast-LIO2 [27]: The baseline algorithm on which our proposed method is based is an advanced, tightly coupled LIO (LiDAR-inertial odometry) system.

All algorithms were executed using their default parameter configurations from the open-source code and ran on the same computer.

### 4.3. Results and Discussion

This section characterizes the GC-LIO algorithm through comprehensive benchmarking and analytical validation on both a self-collected corridor dataset and the public M2DGR dataset. The self-collected dataset is used to verify the algorithm’s ability to suppress vertical and attitude drift in a structured environment with strong planar constraints, in direct comparison with the baseline Fast-LIO2. The M2DGR dataset is used to further evaluate localization accuracy and mapping quality in large-scale, complex scenarios against multiple mainstream LiDAR-based SLAM algorithms. Furthermore, an ablation experiment is conducted to analyze the practical effectiveness and contribution of the coplanarity checking module in the proposed method.

#### 4.3.1. Analysis of Accuracy

To evaluate the effectiveness of the proposed ground constraint in environments with strong planar geometry, we first conducted experiments on the self-collected long-corridor dataset described in Section 4.1.1. Only Fast-LIO2 was used as the baseline for direct comparison, as it is the underlying framework of GC-LIO and ensures that performance differences are solely attributable to the proposed improvements.

**Figure 7:**
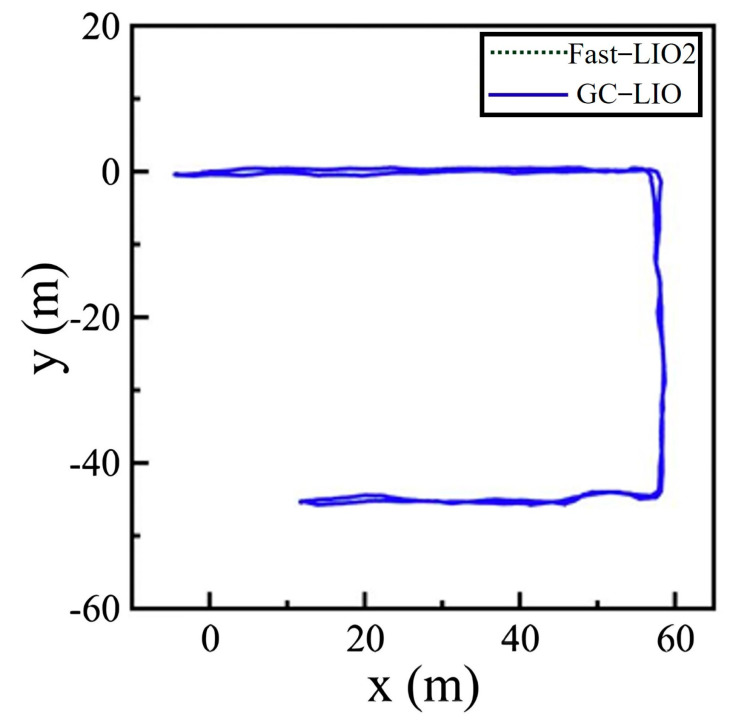
Trajectory diagrams of each algorithm in the xy direction in the corridor dataset.

Figure 7 shows the experimental scene and the top-down XY trajectories generated by both algorithms. The two trajectories almost completely overlap in the horizontal plane, indicating that the proposed ground constraint does not adversely affect horizontal pose estimation.

**Figure 8:**
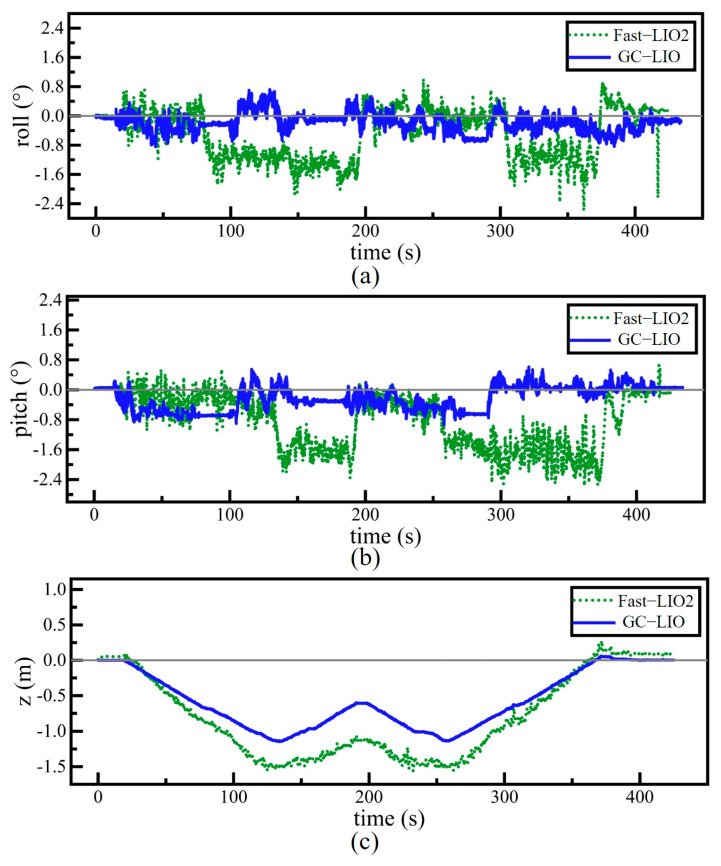
Corridor dataset: diagrams showing the changes in pose estimated by each algorithm over time. (**a**) roll angle variation over time graph. (**b**) pitch angle variation over time graph. (**c**) Z-axis variation over time graph.

As shown in Figure 8, compared to Fast-LIO2, GC-LIO has a terminal cumulative error closer to zero in the roll, pitch, and z dimensions. Additionally, it can be observed that the GC-LIO algorithm’s curve has fewer “spikes” in the figure and a smoother trajectory. This is because GC-LIO introduces more robust ground plane observations in these three dimensions, resulting in less noise compared to the radar point cloud registration observations relied upon by Fast-LIO2.

In addition, in order to more accurately quantify the performance of the ground constraint module in mitigating drift issues, more data sequences were collected within the above corridor scene, and the error values of the z-axis estimates at the endpoints were compared between the two algorithms (since the data collection was closed, the true value can be considered 0), resulting in Table 4.

Table 4 summarizes the z-axis estimation errors of the two algorithms at the end of the self-collected corridor dataset. The baseline algorithm Fast-LIO2 exhibits a noticeable vertical offset relative to the ground truth endpoints, with an average error of 0.0734 m, reflecting the cumulative effect of drift in the absence of explicit planar constraints. In contrast, the proposed GC-LIO algorithm achieves an average z-axis estimation error of 0.0104 m, reducing endpoint errors by approximately 85.83%. This result further confirms that ground constraints can effectively suppress vertical drift in environments with strong planar geometric structures.

**Figure 9:**
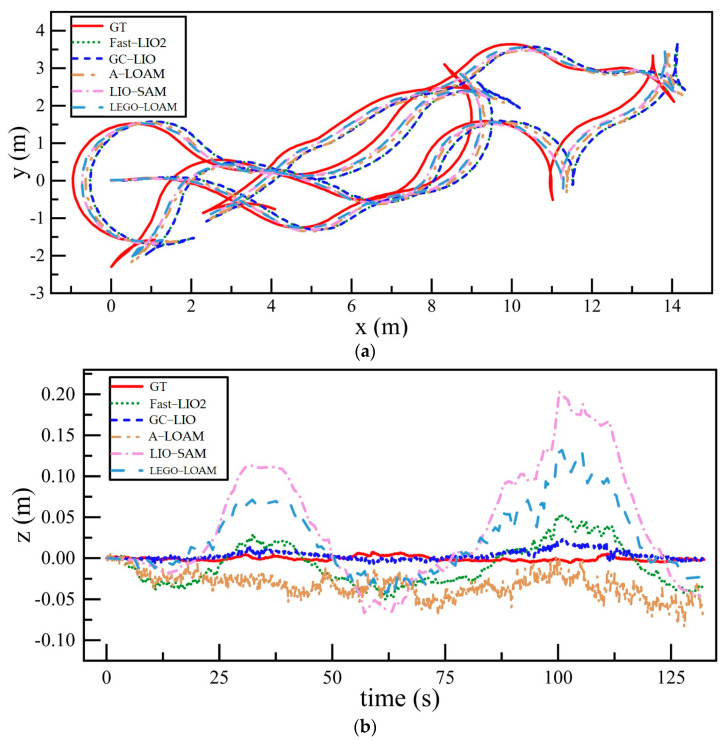
Comparison of trajectories among algorithms. (**a**) The trajectories of all algorithms on the Hall04 sequence compared with the ground truth in the xy direction. (**b**) The trajectory of the algorithm compared with the ground truth on the Hall04 sequence in terms of z-axis variation over time.

To intuitively compare the localization performance of different algorithms, we first conducted tests on the Hall04 sequence of the M2DGR dataset and visualized the trajectories generated by each algorithm against the ground truth. Figure 9 presents the test results for each algorithm.

Figure 9a,b, respectively, show the trajectory comparison results of different algorithms on the Hall04 sequence in the xy-plane and along the z-axis. As clearly seen in Figure 9a, the trajectories of our method (GC-LIO) and the baseline method (Fast-LIO2) on the xy-plane are almost completely overlapping. This is fully consistent with our design expectation, as the core improvement of the algorithm proposed in this chapter—the ground constraint module—primarily constrains the three vertical degrees of freedom in the pose estimation: roll, pitch, and z-axis. It does not directly affect the pose estimation in the xy-plane. Additionally, we observe that although the trajectories of all algorithms generally match the shape of the ground truth in the xy direction, there are still differences in terms of scale and extent of the trajectory, leading to discrepancies between the estimated and true trajectories. This is likely due to the corridor-like structure of the Hall04 environment, which lacks sufficient geometric constraints in the horizontal direction, causing a certain degree of degradation in the performance of all algorithms.

In contrast, the trajectory plot along the z-axis (Figure 9b) clearly demonstrates the superiority of the proposed algorithm. Traditional algorithms such as LIO-SAM and LEGO-LOAM exhibit severe accumulated drift, with their z-axis estimates increasingly deviating from the ground truth (represented by the red solid line) over time. Although the baseline algorithm Fast-LIO2 performs relatively well, it still shows noticeable drift to the naked eye. In sharp contrast to all the comparison algorithms, the z-axis trajectory of the proposed GC-LIO algorithm closely follows the true trajectory throughout the entire operation period. This intuitively proves that our method significantly mitigates the drift issue in pose estimation along the z-axis.

**Figure 10:**
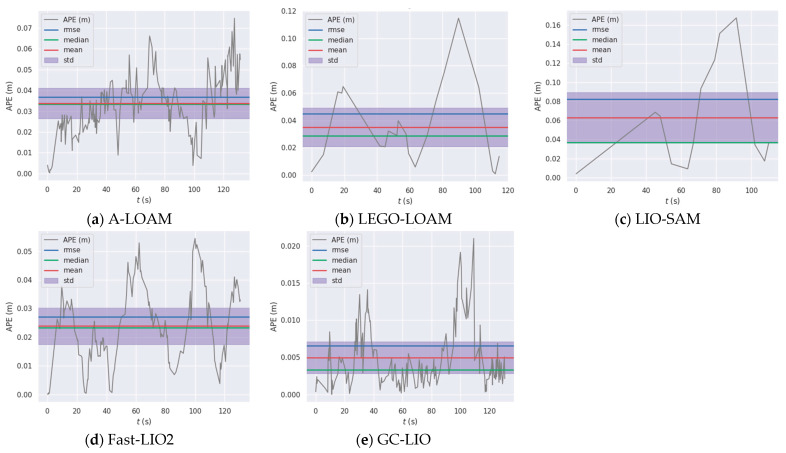
Absolute pose errors of all algorithms in the z-axis direction.

**Figure 11:**
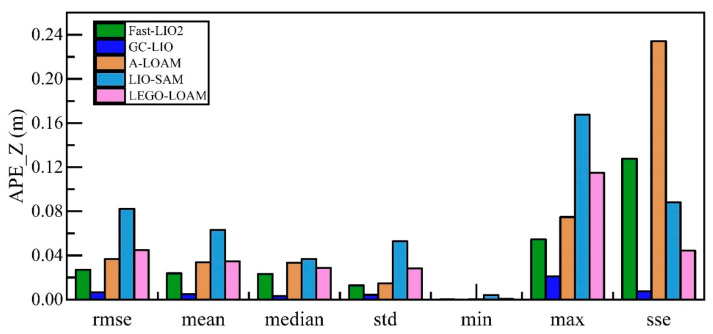
Statistical metrics of absolute pose errors along the z-axis for all algorithms.

Vertical positioning precision is quantified via the z-axis component of APE across evaluated SLAM methods. The detailed statistical data of the absolute pose errors along the z-axis for all algorithms are shown in Table 5, and a visualization of these results is presented in Figure 10 and Figure 11.

**Figure 12:**
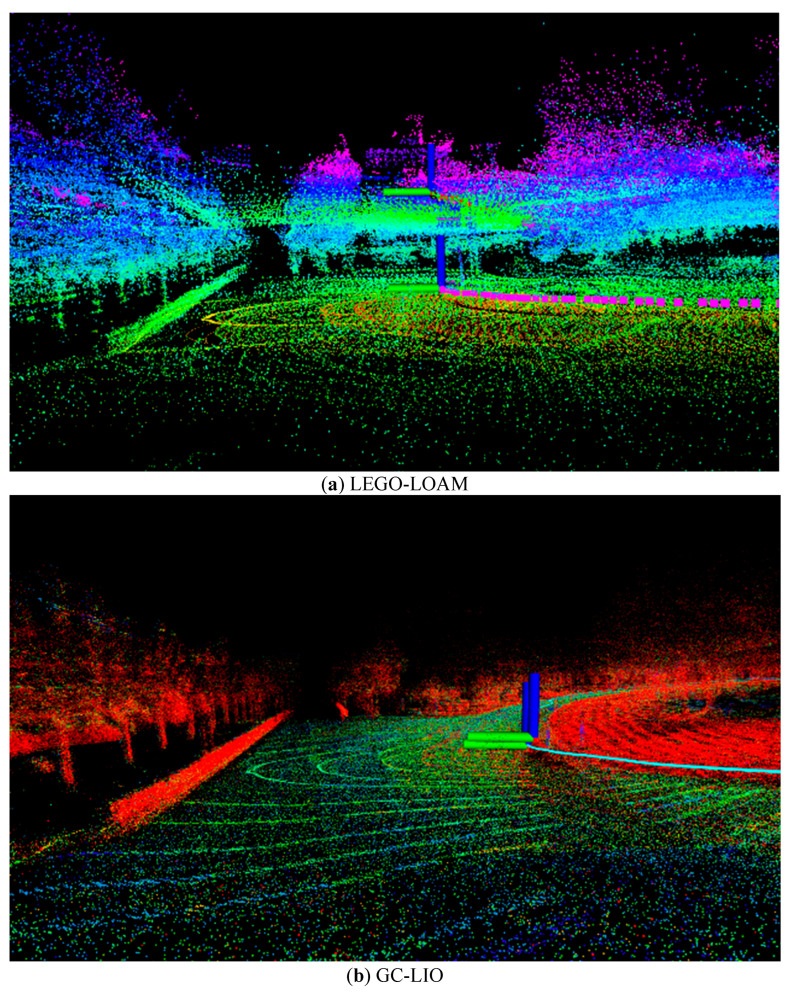
Side view of point cloud maps constructed by different algorithms.

As can be seen from the detailed data in Table 5 and the visual results in Figure 10 and Figure 11, the proposed method GC-LIO significantly outperforms all other algorithms across all statistical metrics. Specifically, as shown in Table 5, the z-axis APE root mean square error (RMSE) of GC-LIO is only 0.007 m, while the corresponding error of the baseline algorithm Fast-LIO2 is 0.027 m. This set of quantitative results irrefutably demonstrates that the ground constraint method proposed in this paper effectively mitigates the vertical pose drift commonly present in traditional SLAM algorithms, thereby achieving higher localization accuracy. Additionally, the improvement in localization accuracy directly contributes to enhanced mapping quality. To effectively evaluate the mapping performance of our algorithm, we conducted mapping tests on the Street04 sequence, which contains loop closures. As shown in Figure 12, based on the high-precision localization results, the map constructed by our algorithm (GC-LIO) exhibits a flat ground surface and consistent loop closure. In contrast, LEGO-LOAM accumulated significant vertical pose drift during operation, leading to a noticeable “double-ground” artifact at the loop closure in the generated map, indicating poor closure performance. This further validates the overall practical advantages of our algorithm in real-world applications.

**Figure 13:**
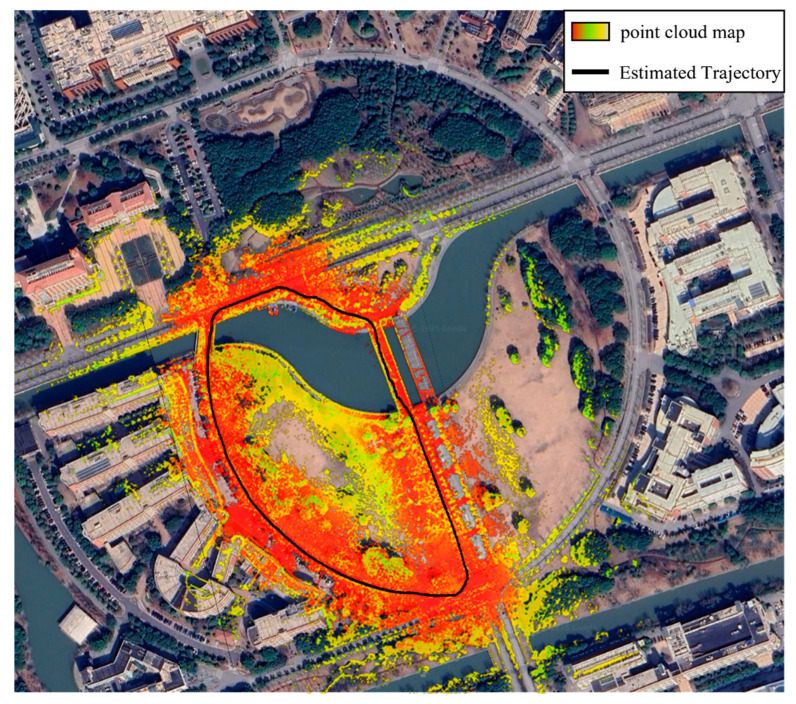
Satellite overhead view of the sequence Street04 with generated point cloud map and robot trajectory.

To holistically validate GC-LIO’s performance in expansive outdoor environments, we geographically align its point cloud maps and trajectories with satellite imagery using the Street04 dataset, as visualized in Figure 13. From the figure, we can clearly see that the point cloud map generated by the algorithm (the colored part in the figure) is highly consistent with the roads, building outlines, and vegetation areas in the satellite image, which indicates that the map is georeferenced accurately. Meanwhile, the black motion trajectory closes the loop well, and the global path is accurate. This result intuitively proves that the algorithm proposed in this study can effectively suppress the cumulative drift under long-time operation, and construct high-quality 3D maps that are globally consistent and accurately aligned with the real world, which is of great application value for urban infrastructure inspection and digital modeling.

#### 4.3.2. Ablation Experiment

To independently verify the effectiveness and necessity of the coplanarity judgment module in our proposed method, we designed an ablation experiment. In this experiment, we ran three versions of the algorithm on the Street04 sequence:

1. The complete version of our proposed method (GC-LIO);
2. A variant of our method with the coplanarity judgment module disabled (referred to as GC-LIO w/o CPJ);
3. The baseline algorithm Fast-LIO2.

**Figure 14:**
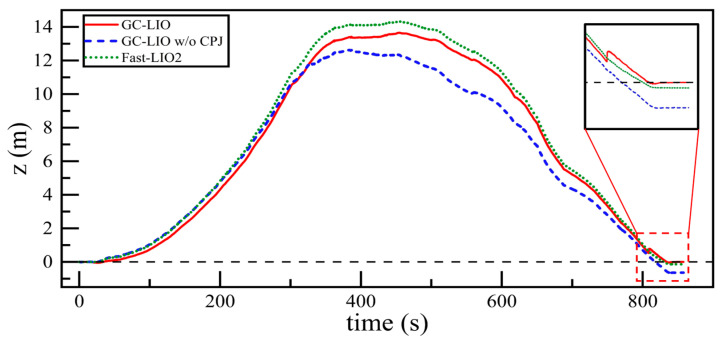
The variation of trajectories along the z-axis over time for all algorithms compared with the ground truth.

We then compared the trajectories of these three algorithms along the z-axis against the ground truth. The results are shown in Figure 14.

As observed from the trajectory curves in Figure 14, the complete GC-LIO algorithm, when operating for approximately 800 s, brings the robot back to a position close to the starting point. At this moment, the algorithm successfully identifies that the current ground plane is coplanar with the reference main plane established at the initial stage, and accordingly activates the ground constraint. As shown in the inset magnified view, the introduction of this constraint rapidly pulls the accumulated z-axis error back toward zero, achieving an almost perfect vertical loop closure.

In contrast, the GC-LIO w/o CPJ variant, which disables the coplanarity judgment module, blindly applies ground constraints throughout the entire operation. This erroneous constraint not only fails to correct the estimation errors but also introduces harmful information into the filter. Consequently, the resulting pose closure error becomes significantly larger, and its performance even deteriorates compared to the unmodified baseline algorithm Fast-LIO2.

The results of this ablation study strongly demonstrate that the proposed coplanarity judgment module is both critical and effective. It ensures that ground constraints are introduced only under safe conditions where the geometric assumption holds true, thereby preventing erroneous constraints from interfering with the system. This significantly enhances the robustness and reliability of the algorithm in real-world, dynamic environments.

## 5. Conclusions and Future Work

This paper proposes a multi-sensor fusion localization and mapping algorithm based on an improved error-state Kalman filter to address the demand for high-precision positioning and mapping in intelligent maintenance of urban infrastructure. Specifically, by introducing a ground point cloud segmentation method based on angular threshold and a dynamic coplanar constraint module, this work effectively integrates local planar consistency of the scene, significantly suppressing pose estimation drift in the vertical direction commonly observed in traditional SLAM algorithms. This study validates the effectiveness of scene prior knowledge combined with multi-sensor fusion strategies in complex environments, providing technical support for high-precision geometric modeling of urban infrastructure.

Although the proposed method demonstrates satisfactory performance in typical urban infrastructure scenarios, the following limitations require further investigation:

1. In scenarios with abrupt ground elevation changes or continuously varying slopes, the current static ground constraint module may experience degraded positioning accuracy due to failure of the local planar assumption.
2. The global map consistency optimization capability of the existing algorithm requires further improvement, along with enhanced robustness against dynamic obstacles.
3. In addition, it should be noted that hardware selection has a non-negligible influence on the experimental results. The LiDAR sensors used in this work have relatively sparse vertical resolution, which directly affects the strength of vertical geometric constraints and may limit performance in certain environments. While our proposed ground constraint mitigates this weakness, sensors with denser vertical channels could further improve accuracy. Moreover, the IMU precision and synchronization quality also contribute to the overall stability of the system. Regarding field tests, the evaluation scenarios—although representative—mainly feature structured environments with sufficient planar regions. More diverse and unstructured field conditions should be considered in the future to comprehensively assess robustness and generalization.

Future research could develop multi-ground principal plane constraints based on sliding windows to enhance the algorithm’s practicality in complex scenarios.

## Figures and Tables

**Table 1:** LiDAR specification parameters.

Specifications	Parameters
Dimensions	∅103×72 m
Rotation Rate	10 Hz
Accuracy	±3 cm
Horizontal field of view	360°
Vertical field of view	30° (+15° to −15°)
Vertical angular resolution	2°
Horizontal angular resolution	0.1°−0.4°
Measurement range	Up to 100 m

**Table 2:** IMU specification parameters.

Specifications	Parameters
Dimensions	∅103×72 m
Sampling Rate	Up to 1 kHz
Accelerometer resolution	0.001 g
Gyroscope resolution	0.001°/s
Weight	78 g
Interface	USB, RS-232

**Table 3:** M2DGR dataset platform sensor parameters.

Transducers	Model	Key Parameters
LiDAR	Velodyne VLP-32C	Horizontal Field of View (H-FoV): 360°, Vertical Field of View (V-FoV): −30° to +10°, Rotation Rate: 10 Hz, Max Range: 200 m, Ranging Accuracy: 3 cm, Horizontal Angular Resolution: 0.2°
RBG Camera	FLIR Pointgrey CM3-U3-13Y3C-CS	Resolution: 1280 × 1024, H-FoV: 190°, V-FoV: 190°, Frame Rate: 15 Hz
GNSS	Ublox M8T	System: GPS/BeiDou, Sampling Rate: 1 Hz
Infrared Camera	PLUG 617	Resolution: 640 × 512, H-FoV: 90.2°, V-FoV: 70.6°, Frame Rate: 25 Hz
VI Sensor	Realsense d435i	RGB/Depth Resolution: 640 × 480, H-FoV: 69°, V-FoV: 42.5°, Frame Rate: 15 Hz, IMU: 6-axis, 200 Hz
Event Camera	Inivation DVXplorer	Resolution: 640 × 480, Frame Rate: 15 Hz
IMU	Handsfree A9	Axes: 9-axis, Sampling Rate: 150 Hz
GNSS-IMU	Xsens Mti 680 G	GNSS-RTK, Localization Precision: 2 cm, Sampling Rate: 100 Hz, IMU: 9-axis, 100 Hz
Laser Scanner	Leica MS60	Localization Precision: 1 mm + 1.5 ppm
Motion-capture System	Vicon Vero 2.2	Localization Accuracy: 1 mm, Sampling Rate: 50 Hz

**Table 4:** Z-axis estimation error values in meters at the endpoints of each algorithm in the corridor dataset.

Algorithm	Sequence				
	01	02	03	04	05
Fast-LIO2	0.027	0.068	0.094	0.076	0.102
GC-LIO	0.007	−0.002	0.013	−0.003	0.027

**Table 5:** Experimental results of the absolute trajectory error in the z-axis direction for each algorithm in the Hall04 sequence.

Algorithm	Error Type					
	RMSE	Mean	Median	Min	Max	SSE
Fast-LIO2	0.027	0.024	0.023	0.000	0.055	0.128
A-LOAM	0.036	0.034	0.033	0.000	0.075	0.234
LIO-SAM	0.082	0.063	0.037	0.004	0.167	0.088
LEGO-LOAM	0.045	0.035	0.029	0.001	0.115	0.044
GC-LIO	0.007	0.005	0.003	0.000	0.021	0.007

## Data Availability

The data presented in this study were derived from the following resources available in the public domain: https://github.com/SJTU-ViSYS/M2DGR (accessed on 10 February 2025).
